# Efficacy and safety of gepants in migraine management: a narrative review focusing on atogepant and rimegepant

**DOI:** 10.22514/jofph.2026.046

**Published:** 2026-07-12

**Authors:** Audrey Gautier, Thomas Gicquel, Marion Mercerolle, Brendan Le Daré

**Affiliations:** ^1^Rennes University, 35000 Rennes, France; ^2^INSERM, INRAE, Institut NuMeCan (Nutrition, Metabolism and Cancer), Rennes University, 35033 Rennes, France; ^3^Biological and Forensic Toxicology Laboratory, Rennes University Hospital, 35033 Rennes, France; ^4^Pharmacy Department, Rennes University Hospital, 35033 Rennes, France

**Keywords:** Migraine, Gepants, Atogepant, Rimegepant, CGRP, Efficacy, Safety

## Abstract

Gepants, oral antagonists of the calcitonin gene-related peptide (CGRP) receptor, represent a new therapeutic class in migraine management. This review aims to assess the efficacy and safety profile of the two gepants currently available in France: atogepant and rimegepant. A review of the literature was conducted in July 2024 using the PubMed, Cochrane Library, and Web of Science databases. Randomized controlled trials evaluating the efficacy and/or safety of atogepant and rimegepant in adult patients with migraine were included. Sixteen randomized controlled trials were selected (nine evaluating atogepant and seven evaluating rimegepant). In migraine prevention, atogepant significantly reduced the mean number of monthly migraine days (−0.7 to −2.4 days *vs.* placebo), with an improvement in quality-of-life scores (Migraine-Specific Quality of Life questionnaire—Role Function-Restrictive domain (MSQ-RFR): +9.9 to +10.8 points). Rimegepant, administered every other day, achieved a smaller reduction (−0.8 days/month) but was comparable to that observed with some anti-CGRP monoclonal antibodies. In acute treatment, a single dose of rimegepant 75 mg provided complete pain relief at 2 hours in 31–33% of patients versus 15% with placebo. Adverse events, mainly gastrointestinal (constipation and nausea), were generally mild to moderate, with no signal of hepatic or cardiovascular toxicity. Gepants represent a major therapeutic advance combining clinically meaningful efficacy with a favorable safety profile. Atogepant appears particularly promising for migraine prevention, whereas rimegepant offers an effective and well-tolerated option for acute treatment. Their use in clinical practice remains limited in France due to high cost and restricted reimbursement. Further real-world and long-term studies are needed to better define their place within current migraine management strategies.

## 1. Introduction

Migraine is a common and debilitating neurological condition, affecting nearly 15% of the global population, with a marked predominance in women [1]. It is characterized by recurrent headaches, often associated with symptoms such as nausea, photophobia, and phonophobia, leading to a significant deterioration in patients’ quality of life. Its socioeconomic impact is considerable, due to the direct costs of care and associated productivity losses [2, 3]. The classification distinguishes between episodic migraine, defined as 1 to 14 days of headaches per month, and chronic migraine, characterized by at least 15 days of headache per month, 8 of which meet the migraine criteria [4].

The pathophysiology of migraine remains incompletely understood, but activation of the trigeminovascular pathway is now recognized as a key mechanism. Activation of meningeal nociceptive fibers leads to the release of vasoactive neuropeptides, foremost among which is calcitonin gene-related peptide (CGRP), involved in pain transmission and modulation [4]. The identification of this central role of CGRP has led to the development of treatments targeting this pathway, including CGRP receptor antagonists, grouped under the term “gepants” [5, 6].

In France, two molecules in this class are currently available: atogepant (Aquipta®), indicated for the prevention of migraine in adults who experience at least four days of migraine per month, and rimegepant (Vydura®), indicated for both the acute treatment of attacks, with or without aura, and the prophylaxis of episodic migraine [5, 6]. These treatments are administered orally, thus providing a convenient alternative to parenteral therapies. Other molecules, such as ubrogepant and zavegepant, are marketed in other countries but are not currently available in France [7].

Despite promising clinical results, the use of gepants in routine practice remains limited, mainly due to the fact that they are not reimbursed. However, the French Society for the Study of Migraines and Headaches (SFEMC) emphasizes their value, recommending their use for prophylaxis or acute treatment, while reserving financial coverage for severe and refractory forms after failure of at least two conventional preventive treatments [7].

In this context of rapidly evolving therapeutic strategies and the emergence of these new molecules, it is essential to accurately assess their efficacy and safety data. Although several reviews and meta-analyses have already evaluated the efficacy and safety of gepants, most were conducted from an international perspective and did not specifically address the regulatory status, accessibility, and clinical positioning of these agents in France. In addition, few reviews simultaneously integrate data from preventive and acute indications while discussing unmet clinical needs and economic constraints. The present work aims to provide a structured narrative review of the efficacy and safety of gepants currently available in France, namely atogepant and rimegepant, with a specific focus on their approved indications, clinical trial data, and place in the French therapeutic landscape.

## 2. Methods

### 2.1 Research methodology

This work was designed as a structured narrative review. Although the literature search and study selection followed predefined and reproducible criteria, no protocol registration or quantitative synthesis was performed, and the review does not claim to follow Preferred Reporting Items for Systematic reviews and Meta-Analyses (PRISMA) guidelines for systematic reviews. This literature review was conducted to present the current state of knowledge on the gepants marketed in France, namely atogepant and rimegepant. The bibliographic search was conducted from 22 to 27 July 2024, using the PubMed, Cochrane Library, and Web of Science databases.

The choice of a structured narrative review was motivated by the marked heterogeneity of available studies, including differences in migraine populations (episodic versus chronic migraine), dosing regimens, treatment indications (preventive versus acute), outcome measures, and follow-up durations, which precluded meaningful meta-analyses.

### 2.2 Eligibility criteria

Randomized clinical trials reporting efficacy data and/or the safety profile of atogepant and/or rimegepant were included. Exclusion criteria were predefined:

- Non-original or review articles.

- Language other than French or English.

- Full articles unavailable at the time of the search.

- Failure to meet inclusion criteria.

- Number of participants less than 300.

- Articles not related to migraine treatment.

### 2.3 Research strategy

A search strategy combining keywords and Medical Subject Headings (MeSH) terms was used. The search terms used included the keywords: “rimegepant”, “atogepant”, “efficacy”, and “safety”. The following search equations were used:

● PubMed:

○ (((efficacy, treatment [MeSH Terms]) OR (drug safety [MeSH Terms]))) AND (rimegepant [Title/Abstract]) Filters: Randomized Controlled Trial.

○ (((efficacy, treatment [MeSH Terms]) OR (drug safety [MeSH Terms]))) AND (atogepant [Title/Abstract]) Filters: Randomized Controlled Trial.

● Cochrane:

○ #1 MeSH descriptor: [Treatment Outcome] explode all trees 203082.

#2 MeSH descriptor: [Drug-Related Side Effects and Adverse Reactions] explode all trees 5225.

#3 (rimegepant): ti, ab, kw 151.

#4 (#1 OR #2) AND #3 6.

○ #1 MeSH descriptor: [Treatment Outcome] explode all trees 203082.

#2 MeSH descriptor: [Drug-Related Side Effects and Adverse Reactions] explode all trees 5225.

#3 (atogepant): ti, ab, kw 129.

#4 (#1 OR #2) AND #3 11.

● Web of Science:

○ Rimegepant (22): (TI = (efficacy) OR TI = (safety)) AND TI = (Rimegepant) AND (TI = (meta-analysis) OR TI = (randomized controlled trial)).

○ Atogepant (3): (TI = (efficacy) OR TI = (safety)) AND TI = (Atogepant) AND (TI = (meta-analysis) OR TI = (randomized controlled trial)).

### 2.4 Selection of studies

All articles resulting from this research and published in French or English were referenced in the bibliographic management software Zotero (Corporation for Digital Scholarship, version 6.0.18, https://www.zotero.org/). Duplicates were then identified and removed. Two successive selection stages were carried out based on the eligibility criteria defined above. The first selection was made by reading the titles and abstracts, and the second by reading the full texts. Two independent reviewers carried out this selection. In the event of disagreement, a consensus was reached after discussion.

### 2.5 Data extraction

The following data were extracted: the name of the first author, the title of the article, the objective of the study, the method used, the size of the sample analyzed, the intervention performed (molecule used, dosage, and duration of treatment), the parameters studied, the efficacy results (average number of migraine or headache days per month, number of days with migraine requiring acute treatment, *etc.*) and safety outcomes (adverse effects, discontinuation of treatment due to adverse effects, *etc.*).

For preventive trials, efficacy outcomes were primarily assessed over a 12-week double-blind treatment period, corresponding to the primary endpoint timeframe of pivotal phase 2/3 studies. Monthly migraine days (MMDs) and monthly headache days (MHDs) were generally calculated as the mean change from baseline during the last month of treatment (typically weeks 9–12) and expressed as differences versus placebo.

### 2.6 Methodological quality and risk of bias assessment

Although this work was not designed as a systematic review, a qualitative methodological appraisal of the included studies was conducted to enhance transparency and interpretability of the findings [8]. The assessment focused on key domains commonly used in evidence evaluation, including study design, randomization procedures, blinding, sample size adequacy, definition and consistency of primary endpoints, duration of follow-up, and completeness of safety reporting.

All included studies were randomized controlled trials, predominantly phase 2/3 or phase 3 trials conducted for regulatory purposes. Randomization procedures were clearly described in all pivotal studies, with parallel-group designs and appropriate allocation methods. The majority of trials were double-blind and placebo-controlled; acute treatment studies involving active comparators used double-dummy designs to preserve blinding.

Sample sizes were generally large, with several hundred participants per treatment arm in most phase 3 trials, providing adequate statistical power to detect clinically meaningful differences in primary efficacy endpoints such as monthly migraine days or pain freedom at 2 hours. Primary and secondary endpoints were prespecified, clinically relevant, and consistent across studies, relying on validated migraine-specific outcome measures including MMDs, Headache Impact Test 6 (HIT-6), MSQ, and Activity Impairment in Migraine Diary (AIM-D) scores.

Blinded treatment periods were typically limited to 12 weeks, which is appropriate for regulatory efficacy assessment but restricts conclusions regarding long-term effectiveness. Longer-term data were mainly derived from open-label extension studies and were therefore interpreted with caution.

### 2.7 Data summary

The data collected during the reading of the articles were summarized in an Excel spreadsheet (Version 2016, Microsoft®, Redmond, WA, USA). A qualitative analysis of the selected articles was then performed to summarize the clinical data and reported adverse effects.

## 3. Results

### 3.1 Selection of studies

The search strategy identified 63 articles. Of these, 22 were duplicates. After reading the titles and abstracts, 21 articles were excluded. The selection process then continued with the exclusion of four articles whose full texts were not available, followed by a review of the full articles. Finally, 16 articles were included in this literature review. The search strategy was presented in the form of a flow chart (Fig. 1).

**Fig. 1.** Item selection flowchart.

### 3.2 Study characteristics

The 16 articles selected are randomized clinical trials. Atogepant is studied in 9 of these articles (for the prophylaxis of migraine in adults) and rimegepant in 7 of them (for the treatment of attacks and prevention of episodic migraine in adults). The methodological details of the studies included in this work are presented in **Supplementary Table 1** (Ref. [9, 10, 11, 12, 13, 14, 15, 16, 17]) (atogepant) and **Supplementary Table 2** (Ref. [18, 19, 20, 21, 22, 23, 24]) (rimegepant).

### 3.3 Efficacy

The criteria analyzed included the mean number of headache days per month (MHD), the number of days requiring acute treatment, and the proportion of patients with at least a 50% reduction in the mean number of migraine days (MMDs) over three months. The impact on quality of life was assessed using the HIT-6 (Headache Impact Test) score, the MSQ (Migraine-Specific Quality of Life) questionnaire in the area of functional restrictions (RFR), and the AIM-D (Activity Impairment in Migraine-Diary) score.

#### 3.3.1 Average number of migraine days per month (MMD)

The clinical trials analyzed show a significant reduction in the number of MMDs in patients taking gepants compared to placebo. At the recommended dose of 60 mg once daily, atogepant was associated with a placebo-adjusted reduction in monthly migraine days ranging from −0.7 to −2.4 days. This broad range reflects differences in study populations (episodic versus chronic migraine) and differences in baseline migraine frequency across trials. Other dosages have also been evaluated: an average reduction of −1.2 days for 10 mg/day and −0.9 days for 30 mg/day, while twice-daily regimens resulted in a reduction ranging from −1.4 to −2.7 days for 30 mg twice daily, and approximately −1.3 days for 60 mg twice daily, again compared to placebo [9, 10, 11] (**Supplementary Table 1**).

Rimegepant 75 mg administered every other day resulted in a modest placebo-adjusted reduction of approximately −0.8 monthly migraine days. Furthermore, a comparative study showed no significant difference between rimegepant and galcanezumab, a monoclonal anti-CGRP antibody, in terms of reducing the number of migraine days per month [18, 19] (**Supplementary Table 2**).

#### 3.3.2 Average number of headache days per month (MHD)

At the recommended dose of 60 mg/day, atogepant is associated with a reduction in the average number of headache days of between −0.9 and −2.2 days per month, confirming its prophylactic efficacy. Other dosages also showed benefits: −1.4 days for 10 mg/day, −1.2 days for 30 mg/day, between −1.2 and −2.8 days for 30 mg twice daily, and −1.4 days for 60 mg twice daily [9, 10] (**Supplementary Table 1**).

#### 3.3.3 Use of acute treatments

The use of acute treatments has been evaluated in preventive trials. For atogepant, the 60 mg/day dose resulted in a reduction ranging from −1.1 to −2.6 days per month, although some studies did not reach statistical significance [9, 10]. Other dosages result in reductions ranging from −1.2 days (60 mg twice daily) to −2.8 days (30 mg twice daily) [10, 11] (**Supplementary Table 1**). In comparison, rimegepant 75 mg every other day induces a more modest reduction (−0.2 days per month) [18]. In acute treatment, rimegepant also reduces the need for rescue medication within 24 hours, with an absolute risk difference ranging from −8.9% to −16% versus placebo [20, 21] (**Supplementary Table 2**).

#### 3.3.4 No pain 2 hours after taking the medication

Pain freedom at 2 hours was defined as a complete absence of headache pain, whereas pain relief refers to a reduction from moderate or severe pain to mild or no pain. Administered as a single 75 mg dose, rimegepant was effective in relieving attacks, with an absolute risk difference of 7.6 to 10.4% for pain-free status at two hours compared to placebo [20]. In a dose-response study, the rates of total relief at two hours were 31.4% for 75 mg, 32.9% for 150 mg, and 29.7% for 300 mg, compared with 15.3% for placebo [22]. The lower doses (10 and 25 mg) and the high dose of 600 mg did not demonstrate significant superiority, probably due to interindividual variability (**Supplementary Table 2**). In the dose-ranging study by Marcus *et al*. [22], sumatriptan 100 mg achieved a pain freedom rate of approximately 35% at 2 hours. The study was not powered to formally compare rimegepant with sumatriptan, and no statistically significant difference between active treatments can therefore be concluded.

#### 3.3.5 Other parameters studied

The sustained efficacy of atogepant was confirmed in extended follow-up studies. In a 12-week study, 81.1% of patients who achieved a ≥50% response maintained this level of efficacy at months 2 and 3, and this rate reached 84.7% after 52 weeks in an open-label study [12] (**Supplementary Table 1**).

Quality of life data, assessed using the MSQ-RFR (Migraine-Specific Quality of Life Questionnaire-Role Function-Restrictive), also showed a significant improvement compared to placebo. For rimegepant, the mean gain was +3.5 points [18, 19], with no significant difference compared to galcanezumab (**Supplementary Table 2**). For atogepant, improvement ranged from +9.9 points (10 mg) to +10.1 points (30 mg), and between +5.4 and +8.0 points at the 60 mg dose, depending on the dosing regimen [11, 13]. These results confirm the positive impact of gepants on the quality of life and daily functioning of migraine patients (**Supplementary Table 1**).

### 3.4 Safety profile

The safety profile was assessed by collecting data on adverse events, serious adverse events, and events leading to discontinuation of treatment, supplemented by clinical and biological monitoring, as well as electrocardiograms (ECGs). Finally, suicide risk was assessed using the Columbia Suicide Severity Rating Scale (C-SSRS), which explores potential suicidal thoughts and behaviors.

#### 3.4.1 Frequency of adverse effects

The safety profile data from the studies analyzed are presented in **Supplementary Table 3** (Ref. [9, 10, 11, 14, 15, 16, 17, 18, 19, 20, 21, 22, 23, 24]).

In the studies analyzed, the frequency of adverse events reported with atogepant ranged from 52% to 67%, with no clear relationship to the dose administered [9, 14]. In the placebo groups, this frequency was comparable, ranging from 48.5% to 56.8% [11, 15]. For rimegepant used in prevention, the frequency of adverse events ranges from 20.5% to 36%, which is close to the placebo level (36% on average) [18, 19]. In acute treatment, it is lower, ranging from 13% to 17.1%, similar to the values observed with placebo [20, 23, 24].

#### 3.4.2 Most common side effects

In patients treated with atogepant, the most commonly reported adverse effects are constipation (2–12.9% versus 0.5–3.6% with placebo) and nausea (4.4–13.3% versus 1.8–5% with placebo), in addition to upper respiratory tract infections in 1–10.3% of cases (compared with 2–8% with placebo) [10, 11, 14, 16, 17].

For rimegepant prophylaxis, the most common adverse events include urinary tract infections (≈2% in both groups), nausea (1.4–3% versus 1% with placebo), and nasopharyngitis (1.7–4% versus 2% with placebo) [18, 19]. In acute treatment, rimegepant is sometimes associated with mild urinary abnormalities (proteinuria 1–1.5% versus 1–1.3% with placebo) and urinary tract infections (0.9–1.5% versus 1–1.5%), as well as nausea (0–8% versus 0.4–3%) [20, 24].

#### 3.4.3 Frequency of serious adverse events

Serious adverse events remain rare and are broadly comparable to placebo. For atogepant, their frequency ranges from 0% to 4.4% (compared to 0–1.2% for placebo) [9, 10, 11, 14]. Among these, Ashina *et al*. [14] (2023) reported a few cases of suicidal ideation, while Goadsby *et al*. [10] (2020) described isolated cases of cholecystitis, urethritis, major depression, or paracetamol overdose, with no established causal link. Tassorelli *et al*. [9] (2024) reported cases of ventricular tachycardia and cancers, also considered non-attributable to treatment.

For rimegepant, serious events in prophylaxis are very rare (0.3–1%, versus 1% with placebo), and even more exceptional in acute treatment (0–0.2%, versus 0–0.4%), mainly including one case of severe low back pain [18, 19, 20, 23].

#### 3.4.4 Frequency of adverse events leading to discontinuation of treatment

Discontinuation rates due to adverse events remain low. For atogepant, they range from 1.8% to 8.5%, compared to 1% to 7.1% for placebo [11, 15, 17]. The main reasons include digestive disorders (nausea, constipation, bloating). For rimegepant used for prevention, discontinuation due to adverse effects occurs in 1.4–2% of patients (compared to 1% in the placebo group) [18, 19]. In the study by Schwedt *et al*. [19] (2024), four discontinuations were reported, related to pulmonary embolism, fatigue, migraine, and drowsiness.

## 4. Discussion

Despite the availability of multiple acute and preventive therapies, a substantial proportion of patients remain inadequately controlled or experience poor tolerability. Gepants address several unmet needs, particularly in patients with contraindications to triptans, insufficient response to conventional preventives, or concerns regarding cardiovascular safety. This literature review aimed to evaluate the efficacy and safety profile of gepants marketed in France, namely atogepant and rimegepant. The results confirm the superiority of both molecules over placebo in prevention, with an average reduction in monthly migraine days (MMDs) ranging from 0.7 to 2.4 days for atogepant, depending on the dosage, and approximately 0.8 days for rimegepant. As a treatment for migraine attacks, rimegepant stood out for its rapid relief, with 31% to 33% of patients achieving pain-free 2 hours after taking the drug, compared with 15% in the placebo group. The safety profile is generally favorable, with the most common adverse events being mild to moderate gastrointestinal symptoms, and rates comparable to placebo for most other manifestations.

In terms of efficacy data, atogepant showed a significant reduction in the number of monthly migraine days, estimated at between −0.7 and −2.4 days compared to placebo at a dose of 60 mg/day. These results are consistent with the meta-analysis by Lattanzi *et al.* [25] (2022), which reported an average decrease of −1.16 to −1.20 days for doses between 10 and 60 mg/day. Furthermore, efficacy was shown to be durable: 84.7% of patients maintained a response of ≥50% after 52 weeks of continuous treatment [12]. For rimegepant, prevention translates into a more modest reduction of −0.8 days per month compared to placebo [18], but this effect is comparable to that observed with certain anti-CGRP monoclonal antibodies in indirect studies [26]. In acute treatment, rimegepant has an odds ratio of 1.84 (95% confidence interval (CI) 1.55–2.17) for absence of pain at 2 hours versus placebo [27], indicating efficacy close to that of sumatriptan 100 mg (35% response versus 29.7–31.4% for rimegepant between 75 and 300 mg/day, 2 hours after administration in the study by Marcus *et al*. [22] (2014)).

Analysis of secondary endpoints also appears to confirm the clinical benefit of gepants. The reduction in the use of acute treatments is estimated at between −1.1 and −2.6 days per month for atogepant at 60 mg/day, compared with −0.2 days for rimegepant in prevention. These results suggest a more pronounced benefit of atogepant in prevention, which is consistent with the meta-analysis by Lattanzi *et al*. [25] (2022), where atogepant significantly reduced the number of days requiring acute treatment (−1.3 to −1.4 days compared to placebo). In the acute treatment indication, rimegepant reduces the need for acute treatment within 24 hours, with an absolute risk difference compared to placebo of 8.9% (95% CI 5.3–12.4%) to 15% (95% CI 10.7–19.3%) depending on the study [20, 21].

The impact on patients’ quality of life has also been documented. In the Atogepant for the Prevention of Migraine in Participants with Episodic Migraine (ADVANCE) trial, atogepant resulted in a mean improvement in the MSQ-RFR score of +9.9 to +10.8 points depending on the dose at 12 weeks, compared with a much more modest improvement in the placebo groups [13]. These data confirm that improvement in daily functioning, also measured by the AIM-D (Activity Impairment in Migraine Diary), is significant at doses of 10 mg and above. For rimegepant, the average improvement in the MSQ-RFR is more modest (+3.5 points) but remains significant [19]. These results are consistent with those reported by Popoff *et al*. [26] (2021), who showed that rimegepant induced a greater improvement in quality of life (MSQ version 2 score) compared to erenumab (anti-CGRP receptor antibody), despite similar efficacy in reducing the number of migraine days.

The safety profile observed in this review is generally reassuring. For atogepant, the frequency of reported adverse events ranged from 52% to 67%, compared with 48% to 57% in the placebo groups, with no apparent dose effect [10, 14]. The most common side effects are constipation (2 to 13% versus 0.5 to 3.6% with placebo) and nausea (4 to 13% versus 2 to 5% with placebo), which is consistent with the results of the meta-analysis by Lattanzi *et al*. [25] (2022) reporting that atogepant at doses of 30 and 60 mg/day is associated with an increased risk of constipation (Odd ratio (OR) 5.19 [95% CI 2.0–13.46] and 4.92 [95% CI 1.89–12.79], respectively) [25]. Upper respiratory tract infections (5 to 10%) are also reported frequently, but at rates similar to those observed in the control groups. For rimegepant, safety appears to be better: the frequency of adverse effects in prevention ranges from 20 to 36%, which is comparable to that of placebo, while in acute treatment it ranges from 13 to 17% [18, 20, 24]. The most common events include urinary tract infections (0.9 to 2%), nasopharyngitis (2 to 4%), and nausea (2 to 8%). The increased risk of nausea is also reported in the meta-analyses by Wang *et al*. [27] (2023) (OR 1.44 [95% CI 1.09–1.90]) and Lattanzi *et al*. [25] (2022) (OR 2.73 [95% CI 1.47–5.06]).

Serious adverse effects remain rare, occurring in less than 4% of patients for both molecules, and do not differ significantly from placebo. With atogepant, there have been a few isolated cases of suicidal thoughts [14]. Cases of cholecystitis, ventricular arrhythmia, or cancer have also been reported, without a clearly established causal link [9]. For rimegepant, serious events during acute treatment are reported exceptionally (<0.5%), including one case of severe low back pain [23]. These observations are consistent with the results of international pharmacovigilance: analysis of the VigiAccess and Food and Drug Administration Adverse Event Reporting System (FAERS) databases by Liang *et al*. [28] (2024) did not identify any significant hepatic or cardiovascular risk, although isolated signals concerning gastrointestinal disorders (constipation, nausea), skin disorders (rash, pruritus, alopecia) or musculoskeletal disorders were noted.

Finally, the issue of cardiovascular risk deserves special attention. Randomized trials of gepants have generally excluded patients with a history of major cardiovascular events, but a phase 3 study of ubrogepant, conducted in high-risk patients, did not show an increased risk of adverse cardiovascular events [29]. However, recent observational data suggest that atogepant may have a less favorable profile in certain at-risk populations, warranting increased vigilance in real-world settings. Overall, the absence of a significant hepatotoxic signal represents a notable advance in this pharmacological class [28].

Comparing gepants with other treatment options is also instructive. In the treatment of migraine attacks, gepants offer similar efficacy to lasmiditan (a 5-hydroxytryptamine (serotonin)-1F receptor agonist used to treat migraine), while having fewer adverse effects than lasmiditan and triptans, which could be an advantage in certain patient populations [30].

Overall, the methodological quality of the included randomized controlled trials can be considered high. Most studies were designed in accordance with regulatory standards, with rigorous randomization, adequate blinding, predefined statistical analysis plans, and clinically meaningful primary endpoints. The consistency of efficacy outcomes across multiple trials, different dosing regimens, and both episodic and chronic migraine populations supports the internal validity of the findings. Nevertheless, several limitations should be acknowledged. First, the number of available randomized trials remains limited, with a median follow-up of approximately 12 weeks, precluding a comprehensive assessment of long-term efficacy and safety. Data beyond this period are mainly derived from open-label extension studies, which limit the robustness of conclusions regarding sustained effectiveness. Second, trial populations were highly selected, with frequent exclusion of patients with chronic migraine, psychiatric or cardiovascular comorbidities, or multiple prior treatment failures, thereby restricting the generalizability of the findings to real-world clinical practice. Third, heterogeneity in efficacy endpoints across studies and the absence of objective biomarkers complicate the assessment and comparison of treatment response. The reliance on electronic patient-reported diaries may also introduce reporting bias. Fourth, the lack of head-to-head comparisons with established reference treatments, such as triptans or anti-CGRP monoclonal antibodies, limits the precise positioning of gepants within current therapeutic algorithms and highlights the need for dedicated comparative and real-world studies. Lastly, an additional important consideration is the potential risk of bias related to industry sponsorship. All included trials were funded by pharmaceutical manufacturers, which may influence study design, endpoint selection, and reporting practices. Nevertheless, several factors mitigate this concern. Primary endpoints were prespecified and aligned with regulatory guidance, safety data were systematically collected and reported using standardized definitions, and no consistent signal of selective outcome reporting was observed. Moreover, the reproducibility of efficacy and safety results across independent trials and study populations strengthens confidence in the robustness of the observed effects. Despite these reassuring elements, the relative scarcity of investigator-initiated studies and real-world comparative data remains a limitation, underscoring the need for post-marketing and real-world evidence to further characterize long-term outcomes.

These findings should also be interpreted in the context of current French recommendations. The SFEMC considers gepants as a therapeutic option for migraine prevention and recommends rimegepant for acute treatment, particularly in patients with contraindications or insufficient response to triptans [7]. However, access to these therapies remains limited in France. For economic reasons, the French Health Authority (HAS) restricts reimbursement of atogepant to patients with high-frequency or chronic migraine (≥8 migraine days per month) after failure of at least two conventional preventive treatments, with an assigned ASMR (improvement in actual clinical benefit) level V, while rimegepant has not sought reimbursement approval. Consequently, the high cost of treatment—approximately €250–300 per month for atogepant and €30–40 per dose for rimegepant—represents a major barrier to widespread use in routine practice [7, 31]. Despite these constraints, the clinical relevance of gepants extends beyond national reimbursement frameworks, as their demonstrated efficacy, favorable tolerability profile, and positive impact on quality of life support their use in carefully selected patients with a substantial disease burden. Although access conditions may vary across countries, the core conclusions regarding efficacy and safety are of broad international relevance and may inform therapeutic strategies in other healthcare systems [7, 32, 33, 34].

## 5. Conclusion

This review summarizes the clinical evidence on gepants currently approved in France for migraine management. Atogepant and rimegepant demonstrate a moderate but statistically significant reduction in monthly migraine days in preventive treatment, with associated improvements in patient-reported outcomes and a favorable safety profile. Rimegepant has also shown efficacy in the acute treatment of migraine, providing pain relief and pain freedom with good tolerability, particularly in patients with contraindications or inadequate response to triptans. Although the evidence base relies mainly on short-term randomized trials conducted in selected populations, findings are consistent across studies and support the clinical value of gepants. Access to these therapies remains limited in France due to reimbursement and cost constraints. Further comparative and real-world studies are required to better define the long-term effectiveness, safety, and clinical positioning of gepants within current migraine treatment strategies.

## Supplementary Material

Supplementary material associated with this article can be found, in the online version, at https://files.jofph.com/files/article/2075412053495824384/attachment/Supplementary%20material.docx.


