# Supplementary material for: Efficacy and safety of gepants in migraine management: a narrative review focusing on atogepant and rimegepant
Source: J Oral Facial Pain Headache. 2026 Jul 12;40(4):1–8. doi: 10.22514/jofph.2026.046 (PMC13434139; doi:10.22514/jofph.2026.046)
Supplement: Supplementary file 1 [file Supplementary-material.docx]

Supplementary material

Supplementary Table 1. Summary of key methodological, demographic, and efficacy data from clinical trials of atogepant.

| Methods | Population | Main criteria studied | Efficacity | Reference |
| --- | --- | --- | --- | --- |
| Phase 3b, multicenter, double-blind, placebo-controlled, randomized clinical trial with parallel groups | Patients were randomized (1:1) to receive atogepant 60 mg (n = 157) or placebo (n = 158) once daily for 12 weeks. | Average number of migraine days per month | MMDs compared to placebo  Atogepant 60 mg: −2.4 days [−3.2 to −1.5] (*p* < 0.0001) | [9] |
| Phase 2b/3, multicenter, double-blind, placebo-controlled randomized trial | Patients were randomized (2:1:2:2:1:1) to receive atogepant 10 mg (n = 93), 30 mg (n = 183), 60 mg (n = 186) once daily, or atogepant 30 mg (n = 86), 60 mg (n = 91) twice daily, or placebo (n = 186) for 12 weeks. | Average number of migraine days per month | MMDs compared to placebo  Atogepant 10 mg/day (−1.2 days [−1.9 to −0.4], *p* = 0.024)  30 mg/day (−0.9 days [−1.6 to −0.3], *p* = 0.039)  60 mg/day (−0.7 days [−1.4 to −0.1], *p* = 0.039)  30 mg twice daily (−1.4 days [−2.2 to −0.6], *p* = 0.0034)  60 mg twice daily (−1.3 days [−2.1 to −0.5], *p* = 0.0031) | [10] |
| Phase 3 randomized, parallel-group, double-blind, placebo-controlled, multicenter trial | Patients with chronic migraine were randomized (1:1:1) to receive atogepant 30 mg twice daily (n = 253), atogepant 60 mg once daily (n = 256), or placebo (n = 246) for 12 weeks. | Average number of migraine days per month | MMDs compared to placebo (with/without overuse of rescue treatments)  Atogepant 30 mg: −2.7 days [−4.0 to −1.4] (*p* = 0.33)/−1.7 [−3.5 to 0.2]  Atogepant 60 mg: −1.9 [−3.2 to −0.6] (*p* = 0.46)/−1.6 [−3.5 to 0.3] | [11] |
| Advance: Phase 3 randomized, double-blind, placebo-controlled trial  Long-term safety (LTS) trial: Randomized, multicenter, open-label trial | Advance study: Patients were randomized (1:1:1:1) to receive atogepant 10 mg (n = 214), 30 mg (n = 223), 60 mg (n = 222), or placebo (n = 214) once daily for 12 weeks.  LTS study: Patients experiencing 4 to 14 migraines per month were randomized (5:2) to receive atogepant 60 mg once daily (n = 546) or standard preventive migraine treatment (n = 198). | Advance: assessment of sustained response to treatment at M2 and M3; assessment of response in participants who did not achieve at least a 50% reduction  LTS: assessment of sustained response to treatment from Q2 to Q4 | Advance  Maintenance of a response ≥50%: 70.8% to 81.1%  Maintenance of a response ≥75%: 47.3% to 61.9%  Maintenance of a 100% response: 34.8% to 41.7%  LTS  Maintenance of a ≥50% response: 84.7%  Maintenance of a ≥75% response: 72.6%  Maintenance of a 100% response: 37.8% | [12] |
| Phase 3, multicenter, double-blind, placebo-controlled randomized trial with parallel groups | Patients experiencing 4 to 14 migraines per month were randomly assigned (1:1:1:1) to receive atogepant 10 mg (n = 214), 30 mg (n = 223), or 60 mg (n = 222) or placebo (n = 214). | MSQ (Migraine-Specific Quality of Life) score  AIM-D PDA score assessing the ability to perform daily activities  AIM-D PI score assessing the physical impact of migraines; HIT-6 score | MSQ RFR compared to placebo at S12  Atogepant 10 mg: 9.90 [5.45 to 14.36] (*p* < 0.0001)  Atogepant 30 mg: 10.08 [5.71 to 14.46] (*p* < 0.0001)  Atogepant 60 mg: 10.80 [6.42 to 15.18] (*p* < 0.0001)  AIM-D PDA compared to placebo at S12  Atogepant 10 mg to 60 mg: −1.19 to −3.32  AIM-D PI compared to placebo at S12  Atogepant 10 mg to 60 mg: −1.08 to −2.46 | [13] |
| Phase 3, multicenter, randomized, open-label trial | Patients experiencing 4 to 14 migraines per month were randomized (5:2) to receive either atogepant 60 mg once daily (n = 546) or standard preventive migraine treatment (n = 198). | Average number of migraine days per month (MMDs) and proportion of patients with a reduction in MMDs of ≥50%, ≥75%, and 100% | MMDs (SE)  Atogepant 60 mg (S1 to S4): −3.8 days (0.1)  Atogepant 60 mg (S49 to S52): −5.2 days (0.2)  Reduction in the number of MMDs (S49–S52)  Atogepant 60 mg (S1 to S4): ≥50% (60.4%); ≥75% (37.2%); 100% (20.7%)  Atogepant 60 mg (S49 to S52): ≥50% (84.2%); ≥75% (69.9%); 100% (48.4%) | [14] |
| Phase 3, multicenter, double-blind, placebo-controlled, randomized trial with parallel groups | Patients experiencing 4 to 14 migraines per month were randomized (1:1:1:1) to receive atogepant 10 mg (n = 214), 30 mg (n = 223), 60 mg (n = 222), or placebo (n = 214) once daily for 12 weeks. | Average number of migraine days per month (MMDs) and per week | MMDs S9 to S12  Placebo: −3.0 days  Atogepant: 10 mg (−4.2 days), 30 mg (−4.3 days), 60 mg (−4.4 days)  *p* ≤ 0.0002  MHDs S1 to S4  placebo: −1.4 days  Atogepant 10 mg (−3.2 days), 30 mg (−3.4 days), 60 mg (−3.9 days)  *p* ≤ 0.0001 | [15] |
| Phase 3 randomized, double-blind, placebo-controlled clinical trial with parallel groups | Patients were randomized (1:1:1:1) to receive atogepant 10 mg (n = 222), 30 mg (n = 230), 60 mg (n = 235), or placebo (n = 223) once daily for 12 weeks. | Reduction of at least 25%, 50%, 75%, or 100% in the mean number of migraines per month (MMDs) | Reduction in MMDs ≥50% *vs.* placebo  Atogepant 10 mg: OR, 3.1 [2.1 to 4.6]; *p* < 0.001  Atogepant 30 mg: OR, 3.6 [2.4 to 5.3]; *p* < 0.001  Atogepant 60 mg: OR, 3.8 [2.6 to 5.7]; *p* < 0.001 | [16] |
| Phase 3 randomized, parallel-group, double-blind, placebo-controlled, multicenter trial (142 centers in 16 countries) | Patients with chronic migraine were randomized (1:1:1) to receive atogepant 30 mg twice daily (n = 257), atogepant 60 mg once daily (n = 262), or placebo (n = 259) for 12 weeks. | Average number of migraine days per month | MMDs compared to placebo  Atogepant 30 mg: −2.4 days [−3.5 to −1.3] (*p* < 0.0001)  Atogepant 60 mg: −1.8 days [−2.9 to −0.8] (*p* = 0.0009) | [17] |

Studies are classified by reference number. LTS: Long-term safety; AIM-D: Activity Impairment in Migraine-Diary; PDA: Performance of Daily Activities; PI: Physical Impairment; HIT-6: Headache Impact Test 6; RFR: Role Function-Restrictive domain; SE: Standard Error; MHDs: monthly headache days; OR: odd ratio.

Supplementary Table 2. Summary of key methodological, demographic, and efficacy data from clinical trials of rimegepant.

| Methods | Population | Main criteria studied | Efficacity | Reference |
| --- | --- | --- | --- | --- |
| Phase 2/3, multicenter, double-blind, placebo-controlled randomized trial | Patients were randomized (1:1) to receive rimegepant 75 mg (n = 373) or placebo (n = 374) every other day for 12 weeks. | Average number of migraine days per month | MMDs compared to placebo  Rimegepant 75 mg: −0.8 [−1.3 to −0.3], *p* = 0.0017  Reduction in MMDs ≥50% *vs.* placebo (S9 to S12)  8% [0 to 15], *p* = 0.044 | [18] |
| Phase 4 randomized, double-blind, double-placebo clinical trial with parallel groups | Patients were randomized (1:1) to receive a subcutaneous injection of galcanezumab 120 mg (n = 287) once a month (after a loading dose of 240 mg) and an oral placebo on other days, or rimegepant 75 mg (n = 293) orodispersible and a subcutaneous injection of a placebo once a month for 3 months. | Proportion of participants who experienced a ≥50% reduction in the number of migraine days per month from baseline during the 3-month double-blind treatment period | MMDs (odds ratio of galcanezumab compared to rimegepant)  Galcanezumab: −0.4 [−0.8 to 0.1]  ≥50% reduction in the number of migraines per month (odds ratio of galcanezumab compared to rimegepant)  Galcanezumab: 1.1 [0.8 to 1.4] (*p* = 0.7) | [19] |
| Phase 3 randomized, double-blind, placebo-controlled trial | Patients suffering from migraines were randomly assigned (1:1) to receive a single dose of 75 mg rimegepant (n = 732) or placebo (n = 734) during a migraine attack. | No pain 2 hours after administration; absence of the most bothersome symptom 2 hours after administration | Absence of pain 2 hours after administration (difference in risk compared to placebo)  Rimegepant 75 mg: 10.4 [6.5 to 14.2] (*p* < 0.001) | [20] |
| Phase 3 randomized, double-blind, placebo-controlled clinical trial | Patients experiencing 2 to 8 attacks per month and fewer than 15 headache days per month were randomized (1:1) to receive a single dose of 75 mg rimegepant (n = 538) or placebo (n = 537) during a migraine attack. | No pain 2 hours after administration; absence of the most bothersome symptom 2 hours after administration | Absence of pain 2 hours after administration (difference in risk compared to placebo)  Rimegepant 75 mg: 7.6 [3.5 to 11.8] (*p* = 0.0004)  Absence of pain 2 to 24 hours after administration (difference in risk compared to placebo)  Rimegepant 75 mg: 7.2 [3.4 to 11.1] (*p* = 0.0002) | [21] |
| Randomized, single-dose, double-blind, multicenter, outpatient trial | Patients with moderate to severe migraine were randomly assigned to receive rimegepant 10 mg (n = 85), 25 mg (n = 68), 75 mg (n = 91), 150 mg (n = 90), 300 mg (n = 121), or 600 mg (n = 92) of rimegepant, 100 mg (n = 109) of sumatriptan, or placebo (n = 229). | No pain 2 hours after administration | Absence of pain 2 hours after administration (% of patients, *p*-value *vs.* placebo)  Rimegepant 75 mg (31.4%, *p* = 0.002)  Rimegepant 150 mg (32.9%, *p* < 0.001)  Rimegepant 300 mg (29.7%, *p* = 0.002)  Rimegepant 600 mg (24.4%, *p* > 0.05)  Sumatriptan (35%, *p* < 0.001)  Placebo (15.3%) | [22] |
| Phase 3, multicenter, double-blind, placebo-controlled randomized trial | Patients suffering from 2 to 8 migraines per month were randomly assigned to receive a single dose of 75 mg rimegepant (n = 594) or placebo (n = 592) during a migraine attack. | No pain 2 hours after administration; absence of the most bothersome symptom 2 hours after administration | Absence of pain 2 hours after administration (absolute difference compared to placebo)  Rimegepant 75 mg: 7.6 [3.3 to 11.9] (*p* < 0.001) | [23] |
| Phase 3 randomized, double-blind, placebo-controlled clinical trial | Patients experiencing 2 to 8 attacks per month and fewer than 15 headache days per month were randomized (1:1) to receive a single dose of rimegepant 75 mg (n = 716) or placebo (n = 715) during a migraine attack. | No pain 2 hours after administration; absence of the most bothersome symptom 2 hours after administration | Absence of pain 2 hours after administration (difference in risk compared to placebo)  Rimegepant 75 mg: 9.2 [5.4 to 13.0] (*p* < 0.0001)  Absence of pain 2 to 24 hours after administration (difference in risk compared to placebo)  Rimegepant 75 mg: 7.7 [4.3 to 11.2] (*p* < 0.0001) | [24] |

Studies are classified by reference number. MMDs: monthly migraine days.

Supplementary Table 3. Safety profile data for gepants in selected clinical studies.

| Reference | Sample size | Frequency of AEs | Most common AEs | Serious AEs | AEs leading to discontinuation of treatment |
| --- | --- | --- | --- | --- | --- |
| [9] | 315 | Atogepant 60 mg (52%) *vs.* placebo (54%) | Constipation: Atogepant (10%) *vs.* Placebo (3%)  COVID-19: Atogepant (8%) *vs.* Placebo (10%)  Nausea: Atogepant (7%) *vs.* Placebo (3%)  Nasopharyngitis: Atogepant (5%) *vs.* Placebo (8%) | Atogepant 60 mg (3%) *vs.* placebo (0%) | Atogepant 60 mg (2%) *vs.* placebo (1%) |
| [10] | 834 | Atogepant 10 mg/day (66%), 30 mg/day (63%), 60 mg/day (58%), 30 mg twice daily (61%), 60 mg twice daily (58%) *vs.* placebo (50%) | Nausea: Atogepant 10 mg/day (5%), 30 mg/day (7%), 60 mg/day (12%), 30 mg twice daily (9%), 60 mg twice daily (10%) *vs.* placebo (5%)  Upper respiratory tract infections: Atogepant 10 mg/day (6%), 30 mg/day (8%), 60 mg/day (5%), 30 mg twice daily (7%), 60 mg twice daily (7%) *vs.* placebo (8%) | Atogepant 10 mg/day (1%), 30 mg/day (1%), 60 mg/day (1%), 30 mg twice daily (0%), 60 mg twice daily (0%) *vs.* placebo (1%) | Atogepant 10 mg/day (4%), 30 mg/day (6%), 60 mg/day (3%), 30 mg twice daily (6%), 60 mg twice daily (8%) *vs.* placebo (3%) |
| [11] | 755 | Atogepant 30 mg (55.8%/57.4%) and 60 mg (66.1%/57.8%) *vs.* placebo (48.5%/51.2%) | Constipation: Atogepant 30 mg (12.9%/7.4%) and 60 mg (10.5%/8.9%) *vs.* placebo (2.9%/3.6%)  Nausea: Atogepant 30 mg (8.0%/7.4%) and 60 mg (7.6%/13.3%) *vs.* placebo (2.9%/4.8%) | Atogepant 30 mg (1.2%/2.1%) and 60 mg (2.3%/3.3%) *vs.* placebo (1.2%/1.2%) | Atogepant 30 mg (3.1%/8.5%) and 60 mg (2.9%/4.4%) *vs.* placebo (2.3%/7.1%) |
| [18] | 747 | Rimegepant 75 mg (36%) *vs.* placebo (36%) | Nasopharyngitis: Rimegepant (4%) *vs.* placebo (2%)  Nausea: Rimegepant (3%) *vs.* placebo (1%)  Urinary tract infections: Rimegepant (2%) *vs.* placebo (2%) | Rimegepant 75 mg (1%) *vs.* placebo (1%) | Rimegepant 75 mg (2%) *vs.* placebo (1%) |
| [19] | 580 | Galcanezumab (20.9%) *vs.* Rimegepant (20.5%) | COVID-19: Galcanezumab (4.2%) *vs.* Rimegepant (1.7%)  Nausea: Galcanezumab (1.0%) *vs.* Rimegepant (1.4%)  Fatigue: Galcanezumab (0.7%) *vs.* Rimegepant (1.4%) | Galcanezumab (0%) *vs.* Rimegepant (0.3%) | Galcanezumab (0.7%) *vs.* Rimegepant (1.4%) |
| [20] | 1466 | Rimegepant (13%) *vs.* placebo (11%) | Nausea: Rimegepant (2%) *vs.* Placebo (<1%)  Urinary tract infection: Rimegepant (1%) *vs.* Placebo (1%)  Dizziness: Rimegepant (1%) *vs.* Placebo (1%) | Rimegepant (0%) *vs.* Placebo (0%) |  |
| [21] | 1075 | Rimegepant 75 mg (15.2%) *vs.* placebo (16.4%) | Proteinuria: Rimegepant (1.5%) *vs.* placebo (1.3%)  Nausea: Rimegepant (1.1%) *vs.* placebo (2.6%)  Urinary tract infections: Rimegepant (0.9%) *vs.* placebo (1.5%) | Rimegepant (0%) *vs.* Placebo (0.2%) |  |
| [22] | 799 |  | Nausea: Rimegepant 10 mg (1.4%), 25 mg (0%), 75 mg (3%), 150 mg (3%), 300 mg (4%), 600 mg (8%), sumatriptan (2%), placebo (2%) | None related to treatment | None |
| [14] | 744 | Atogepant 60 mg (67%) *vs.* active control (78.6%) | Upper respiratory tract infection (10.3%)  Constipation (7.2%)  Nausea (6.3%)  Urinary tract infections (5.2%) | Atogepant 60 mg (4.4%) *vs.* standard care group (3.6%) | Atogepant 60 mg (5.7%): nausea, fatigue, dizziness, rash  group receiving standard care (2.6%) |
| [15] | 873 | Atogepant (52.2%–53.7%) *vs.* placebo (56.8%) |  |  | Atogépant (1.8–4.1%) *vs.* placébo (2.7%) |
| [23] | 1186 | Rimegepant (17.1%) *vs.* placebo (14.2%) | Nausea: Rimegepant (1.8%) *vs.* Placebo (1.1%)  Urinary tract infection: Rimegepant (1.5%) *vs.* Placebo (1.1%) | Rimegepant (0.2%) *vs.* Placebo (0.4%) |  |
| [24] | 1431 | Rimegepant 75 mg (16%) *vs.* placebo (17%) | Proteinuria: Rimegepant (1%) *vs.* Placebo (1%)  Nausea: Rimegepant (1%) *vs.* Placebo (3%)  Urinary tract infections: Rimegepant (1%) *vs.* Placebo (1%) | Rimegepant (<1%) *vs.* Placebo (<1%) |  |
| [16] | 910 | Atogepant 10 mg (52.9%), 30 mg (52.2%), 60 mg (53.7%) *vs.* placebo (56.8%) | Constipation: Atogepant 10 mg (7.7%), 30 mg (7%), and 60 mg (6.9%) *vs.* placebo (0.5%)  Nausea: Atogepant 10 mg (5.0%), 30 mg (4.4%) and 60 mg (6.1%) *vs.* placebo (1.8%) |  |  |
| [17] | 778 | Atogepant 30 mg (56%) and 60 mg (63%) *vs.* placebo (49%) | Constipation: Atogepant 30 mg (11%) and 60 mg (10%) *vs.* placebo (3%)  Nausea: Atogepant 30 mg (8%) and 60 mg (10%) *vs.* placebo (4%) | Atogepant 30 mg (2%) and 60 mg (3%) *vs.* placebo (1%) | Atogepant 30 mg (5%) and 60 mg (3%): nausea, bloating, constipation  Placebo (4%): migraine |

The nature of serious AEs is presented in the manuscript text, when available in the literature. Studies are classified by reference number. AE: adverse events.
